# Neural Control of Balance During Walking

**DOI:** 10.3389/fphys.2018.01271

**Published:** 2018-09-13

**Authors:** Hendrik Reimann, Tyler Fettrow, Elizabeth D. Thompson, John J. Jeka

**Affiliations:** ^1^Department of Kinesiology and Applied Physiology, University of Delaware, Newark, DE, United States; ^2^Department of Kinesiology, Temple University, Philadelphia, PA, United States; ^3^Department of Physical Therapy, Temple University, Philadelphia, PA, United States

**Keywords:** balance, walking, neural feedback, vision, virtual reality, sensorimotor control

## Abstract

Neural control of standing balance has been extensively studied. However, most falls occur during walking rather than standing, and findings from standing balance research do not necessarily carry over to walking. This is primarily due to the constraints of the gait cycle: Body configuration changes dramatically over the gait cycle, necessitating different responses as this configuration changes. Notably, certain responses can only be initiated at specific points in the gait cycle, leading to onset times ranging from 350 to 600 ms, much longer than what is observed during standing (50–200 ms). Here, we investigated the neural control of upright balance during walking. Specifically, how the brain transforms sensory information related to upright balance into corrective motor responses. We used visual disturbances of 20 healthy young subjects walking in a virtual reality cave to induce the perception of a fall to the side and analyzed the muscular responses, changes in ground reaction forces and body kinematics. Our results showed changes in swing leg foot placement and stance leg ankle roll that accelerate the body in the direction opposite of the visually induced fall stimulus, consistent with previous results. Surprisingly, ankle musculature activity changed rapidly in response to the stimulus, suggesting the presence of a direct reflexive pathway from the visual system to the spinal cord, similar to the vestibulospinal pathway. We also observed systematic modulation of the ankle push-off, indicating the discovery of a previously unobserved balance mechanism. Such modulation has implications not only for balance but plays a role in modulation of step width and length as well as cadence. These results indicated a temporally-coordinated series of balance responses over the gait cycle that insures flexible control of upright balance during walking.

## 1. Introduction

Balancing our body while walking is an activity that humans perform seemingly effortlessly. What makes walking so critical to everyday life is that it serves as a platform for functional behavior. Navigation through complicated environments while performing functional tasks such as obstacle avoidance and object manipulation require a dynamically stable base of support. When such upright stability is compromised, the nervous system must devote cognitive resources just to maintain upright balance, severely limiting other functional behavior (Horak, 2006). Mobility is then merely a question of getting from point A to B without a catastrophic event in the form of a fall.

Failure of balance control during walking is a public health problem of enormous importance, with fall-related injuries such as hip fracture costing the US health care system $20–30B per year (Burns et al., 2016). The risk of falling increases with age (Rubenstein, 2006), and older adults rely more on visual information to maintain balance (Tomomitsu et al., 2013; Franz et al., 2015). While there is some evidence that visual information is used in balance-related assessments of the environment (Proffitt et al., 1995), relatively little is known about how the central nervous system uses visual information to control balance during walking.

Neural control of balance has been studied extensively in standing, using a variety of techniques with quiet unperturbed stance as well as sensory and mechanical perturbations (Peterka, 2002). Despite the vast knowledge gained regarding balance control during standing, such findings do not necessarily translate to balance control during walking. The main reason is the gait cycle. While responses to disturbances during standing follow a short-medium-long latency response pattern over 50–200 ms involving a proximal-to-distal pattern (or vice versa) of muscular activation (Horak and Nashner, 1986), responses to disturbances during walking can occur anytime over the much longer (≈600 ms) gait cycle of steady state walking. Critically, body configuration changes dramatically over the gait cycle (e.g., double vs. single stance), necessitating vastly different mechanisms to maintain upright balance at different points of the cycle.

Here we investigate how healthy young adults respond to visual disturbances while walking on a treadmill in a virtual reality environment. This virtual environment is intermittently manipulated to give visual sensations that are designed to be indistinguishable from the optical flow experienced during an actual lateral fall. Much of the research on human walking has focused on mechanical perturbations. While providing useful insights, this approach has the problem that when observing a system response to a mechanical perturbation, sophisticated techniques must be employed to disambiguate the neural control action from the purely mechanical response of the musculoskeletal system. Sensory perturbations, which have this problem to a much smaller degree, have been used mostly to study other functions during walking, such as the visual system for navigation (Patla and Vickers, 1997), steering (Warren et al., 2001), and speed control (Konczak, 1994; Lamontagne et al., 2007). More recently, interest in the role of the visual system for balance control is expanding from standing to walking (Logan et al., 2010). While responses of the upper body seem to be somewhat similar between standing and walking at low frequencies, gains are higher during walking at high frequencies (Anson et al., 2014). This is no doubt due to the nature of the gait cycle, which has a strong modulating effect on balance responses in the lower body (Logan et al., 2014; Qiao et al., 2018). The details of these responses, however, are currently not well understood.

The locomotion literature has largely focused on changes in foot placement as a means to maintain upright balance during walking (Bauby and Kuo, 2000; Wang and Srinivasan, 2014; Vlutters et al., 2016). The gravitational torque is modulated by shifting the location of the next foot placement in the direction of a perceived fall. This mechanism predicts a modulation of the swing leg hip abductor muscle activation, a change in the hip ab/-adduction angle and a shift of the swing foot heel position in the direction of the perceived fall. However, recent studies have suggested that humans also make systematic use of a lateral ankle roll mechanism to complement foot placement control (Reimann et al., 2017; Hof and Duysens, 2018). Before foot placement changes are initiated, a torque is generated around the stance foot ankle in single stance that corrects the upper body against the fall. This mechanism predicts a modulation of the stance foot ankle everter muscle activation, a change in ankle in-/eversion angle and a shift of the CoP in the direction of the perceived fall.

In the current study, we have uncovered a third mechanism. We observe systematic changes in the modulation of the ankle plantar-/dorsiflexion angle and associated musculature near the end of the double stance phase, making a further contribution to upright balance control. Such modulation has implications not only for balance but plays a role in modulation of step width and length as well as cadence during walking.

## 2. Materials and methods

Twenty healthy young adult subjects (11 female), between 18 and 37 years of age (22.8 ± 4.1), weighing 75.2 ± 17.9 kg, volunteered for this study. Subjects provided informed verbal and written consent to participate. Subjects with self-reported history of neurological disorder or surgical procedures involving the legs, spine or head were excluded. Experiments were performed at Temple University and the design was approved by the Temple University Institutional Review Board.

### 2.1. Experimental design

**Figure 1:**
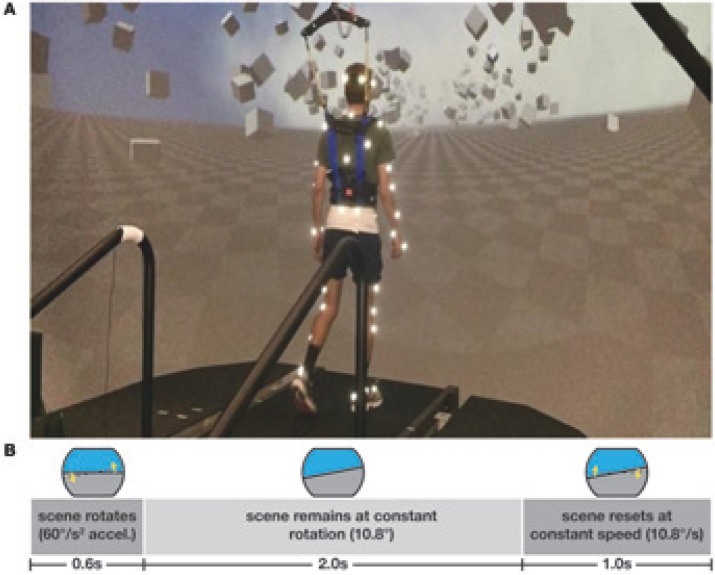
Experimental setup **(A)** and stimulus pattern **(B)**. Subjects walked on a treadmill in the virtual reality environment. Triggered on heelstrike, the environment rotates for 0.6 s, then remains static for 2 s before resetting with uniform speed over 1 s.

Subjects walked on a split-belt treadmill in a virtual environment projected onto a curved dome that covered almost their entire field of vision (Bertec, Inc.). The treadmill was self-paced, using a nonlinear PD-controller in Labview (National instruments Inc., Austin, TX, USA) to keep the markers on the posterior superior iliac spine on the mid-line of the treadmill. The same speed command was sent to each belt of the treadmill. The virtual environment consisted of a tiled marble floor with floating cubes randomly distributed in a volume 0–10 m above the floor, 2–17 m to each side from the midline, and infinitely into the distance, forming a 4 m wide corridor for the subjects to walk through (see Figure 1), implemented in Unity3d (Unity Technologies, San Francisco, CA, USA). Perspective in the virtual world was linked to the midpoint between the two markers on the subject's temples, superposed over forward motion defined by the treadmill speed. We induced visual fall stimuli by rotating the virtual world around the anterior-posterior axis through the midline of the floor. Triggered on heelstrike, the stimulus consisted of a rotation accelerating at 60°*s*^−2^ for 600 ms in a randomized direction. The resulting rotation of 10.8° was then held constant for 2,000 ms, before being reset to neutral rotation with uniform speed over 1,000 ms. After resetting to neutral rotation, a randomized interval of 10–13 steps elapsed before the next stimulus was triggered. Heelstrikes were defined as downward threshold crossings of the vertical heel-marker position. The threshold was set to the vertical heel-marker position of each foot during quiet standing, plus 3 mm.

Reflective markers were placed bilaterally on the feet, lower legs, thighs, pelvis, torso, head, upper arms, forearms, and hands of the subject, using the Plug-in Gait marker set (Davis et al., 1991) with six additional markers on the anterior thigh, anterior tibia, and 5th metatarsal of each foot for a total of 45 markers. Marker positions were recorded at 250 Hz using a Vicon motion capture system with nine cameras. Surface electromyographical data (EMG) was collected from the *gluteus medius, tensor fasciae latae, gastrocnemius medialis, tibialis anterior*, and *peroneus longus* muscles on each leg. Data for the tensor fasciae latae is missing for six subjects because of hardware problems. Ground reaction forces and moments were collected at 1,000 Hz from both sides of the instrumented split-belt treadmill (Bertec, Inc.). Forces and moments were transformed into a common coordinate frame and then used to calculate the whole-body CoP (Winter, 1990).

After explaining the experiment, obtaining consent and placing markers and EMG sensors, subjects first walked for 15 min on the self-paced treadmill in the virtual environment to adapt to this experimental setup. We then stopped the treadmill and told the subjects that we would now perturb their sense of balance by modifying the virtual scene, and asked them to cope with this perturbation “normally” and keep walking forward. Data collection blocks consisted of two alternating phases for *metronome* and *stimulus*. During metronome phases, lasting 30 s, subjects were provided an auditory metronome at 90 bpm and asked to use this as an “approximate guideline” for their footsteps, both during metronome and stimulus phases. During stimulus phases, lasting 120 s, the metronome was turned off, and subjects received visual fall stimuli as described above. Data were collected during stimulus phases. Each subject performed four blocks of walking, each block consisting of five metronome and five perturbed phases, always starting with metronome phases, for a total of 12.5 min per block. After each block, the treadmill was turned off and subjects were offered a break. This protocol was implemented in a custom Labview program that sent the head position, treadmill speed and rotation angle to the Unity computer via UDP and saved the visual rotation angle and treadmill speed at 100 Hz.

### 2.2. Data processing

Kinematic data were low pass filtered with a 4th order Butterworth filter at a cut-off frequency of 10 Hz. Small gaps in the marker data of up to 100 ms length from occlusions were filled using cubic splines. Time points with remaining marker occlusions were excluded from further analysis. From the marker data, we calculated joint angle data based on a geometric model with 15 segments (pelvis, torso, head, thighs, lower legs, feet, upper arms, forearms, hands) and 38 degrees of freedom (DoF). We estimated the hip joint centers based pelvis landmarks following Tylkowski (Tylkowski et al., 1982; Bell et al., 1990), and the knee joint centers and knee flexion rotational axes from reference movements using the symmetrical axis of rotation approach (Ehrig et al., 2007). We performed inverse kinematics by minimizing the distance between the measured and the model-determined marker positions (Lu and O'Connor, 1999). This optimization was performed first for the six pelvis DoFs, which formed the root of the kinematic tree, then for the six DoFs at the lumbar and cervical joints, and last for each of the arms and legs separately. We estimated the body center of mass (CoM) positions based on estimated segment CoM locations (Dumas et al., 2007) and the inverse kinematics and calculated CoM velocities and accelerations using numerical derivation by time.

We identified heelstrike events for each foot by finding negative peaks in the vertical positions of the heel markers with minimal inter-peak distances of 250 ms and peak prominence >2 cm, and pushoff events as the first peak in the vertical velocity of the 2nd metatarsal marker with a prominence >0.35ms^−1^ after each heelstrike. We visually inspected the result of this automatic identification and applied manual corrections in the rare cases where events were mis-identified.

We then partitioned the data into steps, defined as heelstrike to contralateral heelstrike. For each visual stimulus, we identified the four steps following the triggering heelstrike and the two steps preceding it as relevant for further analysis, for a total of six steps per fall stimulus. We used the two steps before each stimulus trigger as unperturbed control steps. For each step, all data was then time-normalized to 100 time points. Steps containing missing kinematic data or where the inverse kinematics optimization method had failed were excluded from further analysis. After removing these steps, a total of 17,457 stimulus steps remained, distributed among the four-steps analysis period. The range of steps in each combination of trigger foot, stimulus direction and post-stimulus step index was 986–1205.

Assuming body symmetry, we classified each combination of triggering foot and fall stimulus direction in a perceived fall *toward* the triggering foot or *away* from it. For the stimulations triggered by the left foot, we inverted the spatial variables, so that positive means toward the triggering foot for all data points. For all trajectories, we subtracted the mean of the control data for the same stance foot from the stimulus data to estimate the response to the stimulus. For the lateral foot placement, we fitted a linear regression model relating the foot placement changes for each subject to the changes of lateral position and velocity of the CoM at midstance using the control data (Wang and Srinivasan, 2014). Then for each stimulus step, we used this model to estimate the expected foot placement change based on the CoM state, and subtracted this from the observed foot placement change, resulting in an estimate of the foot placement change due to the visual stimulus (Reimann et al., 2017). We will refer to this model-based estimate as *stimulus-induced foot placement change*.

To estimate the onset time of a response, we determined a range of interest as the data where the 95% confidence interval around the mean of the pooled data excluded zero. Within that range, we found the first local maximum of the response's rate of change and fitted a linear approximation to the response curve at that point. The intersection of the linear approximation with the zero line was used as an estimate of the onset time for the response (Oostwoud Wijdenes et al., 2014).

Force plate data was low pass filtered with a 4th order Butterworth filter at a cut-off frequency of 50 Hz. EMG data was rectified, then low-pass filtered with a 4th order Butterworth filter at a cut-off frequency of 6 Hz. For each subject and EMG channel, we calculated the average activation across all control strides and used this value to normalize EMG before averaging across subjects.

### 2.3. Statistical analysis

To test our hypotheses about whether humans use the foot placement and the lateral ankle mechanisms to control balance in response to visually induced fall sensations during locomotion, we used R (R Core Team, 2013) and *lme4* (Bates et al., 2014) to perform a linear mixed effects analysis. As fixed effects, we used *triggering foot* (left/right) and *perturbation direction* (toward/away from triggering foot), and a possible interaction between these two. As random effects, we used individual intercepts for subjects.

#### Outcome variables

To test our hypothesis about the foot placement mechanism, we analyzed the following four variables related to the swing leg: *(i) foot placement* is defined as the medial-lateral position of the swing leg relative to the stance leg at heelstrike, and *foot placement change* is the difference between a stimulus *foot placement* and the average of the control *foot placements*. *(ii) stimulus-induced foot placement change* is defined as the difference between the measured *foot placement* value and the *foot placement* value predicted based on the position and velocity of the CoM at mid-stance using the linear model (see above). *(iii) hip abduction change* is defined as the abduction/adduction angle of the swing leg hip at the first post-stimulus heelstrike, with the average over the control steps subtracted. *(iv) integrated gluteus medius EMG change* is defined as the gluteus medius EMG of the swing leg, with the average over the control steps subtracted, then integrated over the first post-stimulus swing phase.

To test our hypothesis about the lateral ankle roll mechanism, we analyzed the following three variables related to the stance leg: *(v) integrated relative CoP change* is defined as the medial-lateral position of the CoP relative to the CoM, with the average over the control steps subtracted, then integrated over the first post-stimulus swing phase. *(vi) ankle eversion change* is defined as the eversion/inversion angle of the stance leg ankle at the first post-stimulus heelstrike, with the average over the control steps subtracted. *(vii) integrated peroneus longus EMG change* is defined as the peroneus longus EMG of the stance leg, with the average over the control steps subtracted, then integrated over the first post-stimulus swing phase.

To allow testing whether the magnitude of the motor response depends upon the direction of the stimulus relative to the stance leg, we inverted the outcome variables depending on perturbation direction. The spatial outcome variables of foot *foot placement change, stimulus-induced foot placement change*,and *integrated relative CoP change* were inverted for perturbation direction *away* and the other, body-relative outcome variables for perturbation direction *toward*. All outcome variables used in the statistical analysis pertained to step ONE after the stimulus. We confirmed the assumptions of normality and homoscedasticity by visual inspection of the residual plots.

#### Significance of effects

For each outcome variable, we fitted a linear mixed model and performed an ANOVA to analyze the effects of *trigger foot* and *perturbation direction*, using Satterthwaite's method (Fai and Cornelius, 1996) implemented in the R-package *lmerTest* (Kuznetsova et al., 2017). To analyze whether the differences between stimulus and control steps represented by the outcome variables are statistically significant, we calculated the least-squares means and estimated the 95% confidence intervals for the intercept of each outcome variable at each level of the significant factor, using a Kenward-Roger approximation (Halekoh and Højsgaard, 2014) implemented in the R-package *emmeans* (Lenth, 2016). Results were judged statistically significant when the 95% confidence interval did not include zero.

We refrained from approximating *p*-values for the ANOVA directly in the traditional format, which can currently not be calculated reliably due to the lack of analytical results for linear mixed models (Bates et al., 2014). As a general note, we limited the statistical tests to the concrete hypotheses that we had prior to performing the experiment (Brenner, 2015), but did not perform formal statistical tests on effects that we discovered in the exploratory part of the experiment. While proper testing of these effects will be a subject of further study, we point out that many of the observed patterns that we report and discuss below appear with a high degree of symmetry in the data for both perturbation directions, which can serve as an indicator of their reliability.

#### Visualization

The method for estimating confidence intervals described above requires fitting a linear model, which is too time-consuming to be feasible for all available data. For visualization purposes, we calculated the 95% confidence interval at each normalized time point for all reported trajectories using the assumption that all data at that time point is independent and normally distributed.

#### Power analysis

We performed a power analysis on the first three subjects using *SIMR* (Green and Macleod, 2016) with 100 simulations to determine the required sample size. For all seven outcome variables, we estimated the available power for *N* = 3 and, where necessary, the number of subjects required to reach 95% power for an effect size deemed functionally meaningful (see Table 1). While the results indicated that 14 subjects would provide sufficient power for all seven outcome variables, we decided to collect 20 subjects in total to better meet our secondary aim of exploring the details of balance control beyond the already anticipated effects.

**Table 1:** Results of the power analysis.

**Variable**	**Δ_rel_**	**Power at *N* = 3 (%)**	***N* required for 95% power**
Foot placement change	5 mm	98	–
Stimulus-induced foot placement change	5 mm	100	–
Hip abduction change	1 deg	100	–
Integrated gluteus medius EMG change	1 % s	38	13
Integrated relative CoP change	1 mm s	100	–
Ankle eversion change	1 deg	100	–
Integrated peroneus longus EMG change	2 % s	41	14

*Δ_rel_ is the effect size deemed functionally relevant*.

## 3. Results

**Figure 2:**
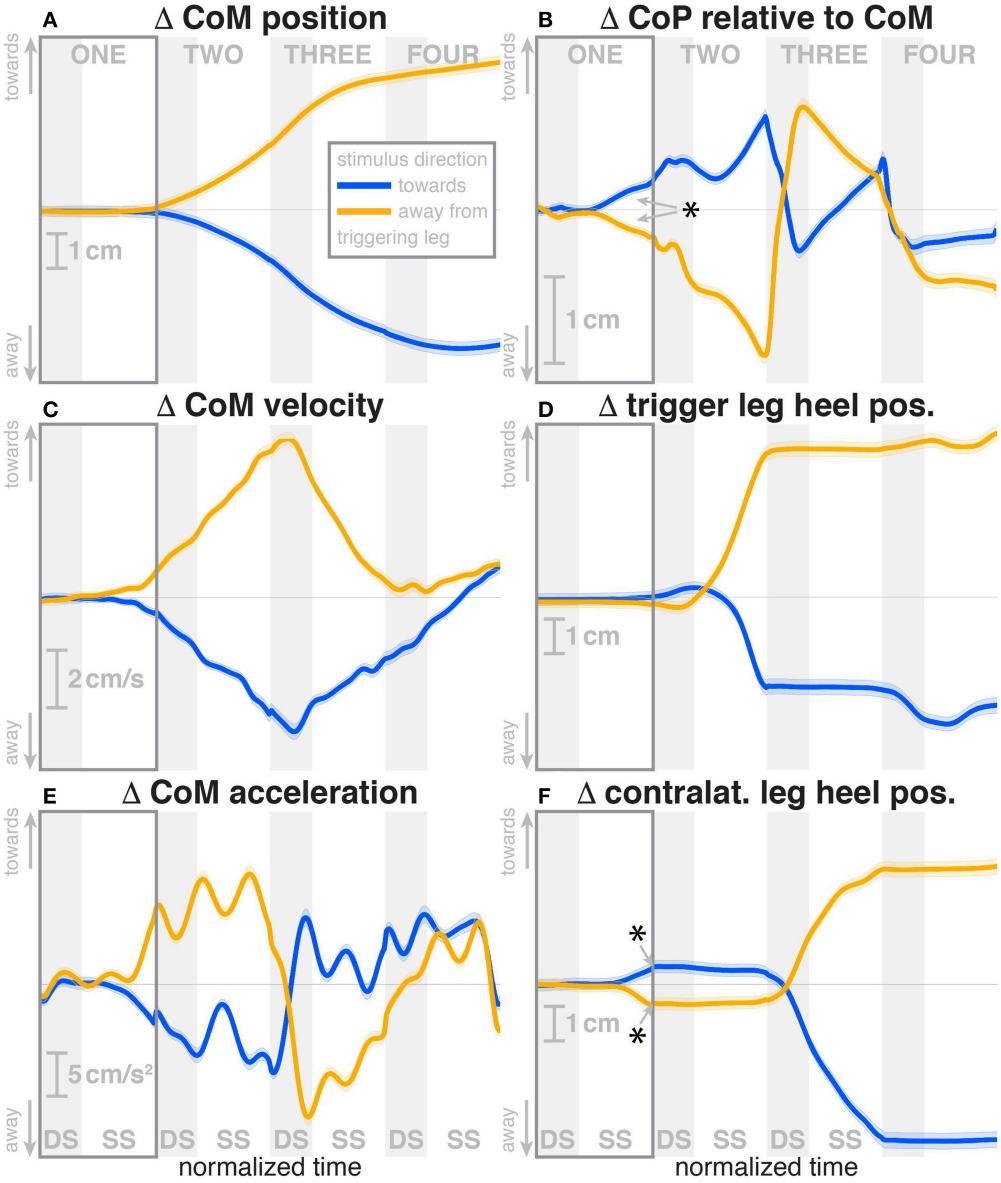
Changes in response to the visual stimulus in the medial-lateral CoM kinematics **(A,C,E)**, CoP **(B)**, and heel positions **(D,F)**. The baseline represents the average of the control steps. Curves start at the heelstrike triggering the stimulus and show the subsequent four steps in normalized time, with the first step marked for clarity. Blue curves correspond to fall stimuli *toward* the triggering leg, orange curves to fall stimuli *away* from the triggering leg. Asterisks indicate statistically significant difference from zero (see **Table 3**). Lack of marks indicate that no hypothesis was statistically tested.

Subjects were able to cope with the visual fall stimuli without making use of the harness or stepping off the treadmill. Subjects' CoM swayed laterally in the direction opposite to the perceived fall, i.e., when the perceived fall was *toward* the triggering leg, the CoM moved *away* from it, as illustrated in Figure 2. The peak average CoM excursion is 4.1 cm at the end of the fourth step. The Average CoM velocity peaked during the double stance of the third post-stimulus step and tended toward zero by the end of the fourth step.

The ANOVA revealed that the *trigger foot* (left/right) had no statistically significant effect on any outcome measure, but the effect of *perturbation direction* (toward/away from the trigger foot) was statistically significant for two of the seven outcome measures (see Table 2). Based on these results, we report the outcomes as averages across the two trigger foot conditions, but separated by stimulus direction.

**Table 2:** Results of the ANOVA indicating which factors have a significant effect on the *magnitude* of the motor response to the visual stimulus (see Table 3 for statistics on the *existence* of the motor responses).

		**Numerator Df**	**Denominator Df**	***F***	***p***
Foot placement change	Trigger foot	1	4429	2.48764	0.1148
	Direction	1	4432	0.07423	0.7853
	Trigger foot^*^direction	1	430	1.33762	0.2475
Stimulus-induced foot placement change	Trigger foot	1	4424	0.14051	0.7078
	Direction	1	4425	21.8447	<**0.0001**
	Trigger foot*direction	1	4424	0.01517	0.9020
Hip abduction change	Trigger foot	1	4427	1.3841	0.2394
	Direction	1	4429	12.4559	**0.0004**
	Trigger foot*direction	1	4427	0.1607	0.6885
Integrated gluteus medius EMG change	Trigger foot	1	4435	0.03125	0.8597
	Direction	1	4440	2.24167	0.1344
	Trigger foot^*^direction	1	4437	0.11431	0.7353
Integrated relative CoP change	Trigger foot	1	4427	0.2691	0.604
	Direction	1	4429	2.1691	0.1409
	Trigger foot^*^direction	1	4427	1.1234	0.2893
Ankle eversion change	Trigger foot	1	4423	0.2205	0.6387
	Direction	1	4424	0.0007	0.9795
	Trigger foot^*^direction	1	4423	1.3456	0.2461
Integrated peroneus longus EMG change	Trigger foot	1	4426	0.02324	0.8788
	Direction	1	4428	0.98114	0.322
	Trigger foot^*^direction	1	4426	1.89504	0.1687

*Numerator and denominator degrees of freedom calculated using Satterthwaite's approximation. Statistically significant effects are marked by bold-face*.

For the detailed results, we first present the immediate neuromotor responses during the first post-stimulus step, and then the secondary responses during the following three steps. For ease of reference, we marked this immediate response period of the first step visually in all data figures. We added asterisks to the figures denoting statistically significant effects, but these are for reference only since there were some differences in data processing for visualization and for statistical analysis. Throughout the results, we refer to the direction of the visual stimulus as *toward* or *away* from the leg that triggered the perturbation, i.e., a *toward*-stimulus is a perceived fall to the right that was triggered by a right heelstrike, or a perceived fall to the left triggered by a left heelstrike. Assuming lateral symmetry, we express the directions of the motor responses in the same reference frame as as toward or away from the triggering leg, instead of spatial directions.

### 3.1. Immediate responses

During the first post-stimulus step, the CoP shifted in the direction of the perceived fall relative to the COM (see Figure 2B). The integrated change over the first single-stance phase is significantly different from zero for both stimulus directions (see Table 3). This shift begins 309 ms after stimulus onset for perceived falls *toward* the triggering leg and 300 ms for perceived falls *away* from it. The trigger leg ankle in-/eversion angle systematically changed with the visual stimulus during step ONE (see Figure **4A**, Table 3). The ankle joint inverted for fall stimuli *toward* the triggering leg and everted for *away* stimuli, as predicted by the lateral ankle mechanism. The onset time of the ankle in-/eversion change was estimated as 351 ms for *toward* and 335 ms for *away* stimuli. The trigger leg *peroneus longus* activity, an ankle evertor, was decreased for *toward* stimuli and increased for *away* stimuli (see Figure **5A**, Table 3). The onset time of this EMG change was estimated as 185 ms for *toward* and 214 ms for *away* stimuli. The direction of this systematic modulation is in accordance with our expectation from the lateral ankle mechanism.

**Table 3:** Least-squares means and upper and lower limits of the 95% confidence intervals for each outcome variable for stimulus *toward* and *away* from the triggering leg using Kenward-Roger approximation.

	***Toward***** (*****N*** = 2225**)**			***Away***** (*****N*** = 2224**)**		
**Variable**	**Mean**	**Lower**	**Upper**	**Mean**	**Lower**	**Upper**
Foot placement change (mm)	**4.342**	**2.093**	**6.59**	**4.041**	**1.792**	**6.29**
Stimulus-induced foot placement change (mm)	**4.145**	**2.777**	**5.514**	**6.022**	**4.653**	**7.39**
Hip abduction change (deg)	0.016	−0.089	0.121	**0.170**	**0.065**	**0.276**
Integrated gluteus medius EMG change (% s)	−0.229	−1.290	0.832	0.837	−0.223	1.898
Integrated relative CoP change (mm s)	**0.459**	**0.114**	**0.805**	**0.675**	**0.330**	**1.021**
Ankle eversion change (deg)	**0.469**	**0.237**	**0.701**	**0.468**	**0.236**	**0.700**
Integrated peroneus longus EMG change (% s)	**2.854**	**0.233**	**5.476**	**3.877**	**1.255**	**6.498**

*Confidence intervals not including zero indicating the existence of a motor response to the visual stimulus in a variable are marked by bold-face*.

**Figure 3:**
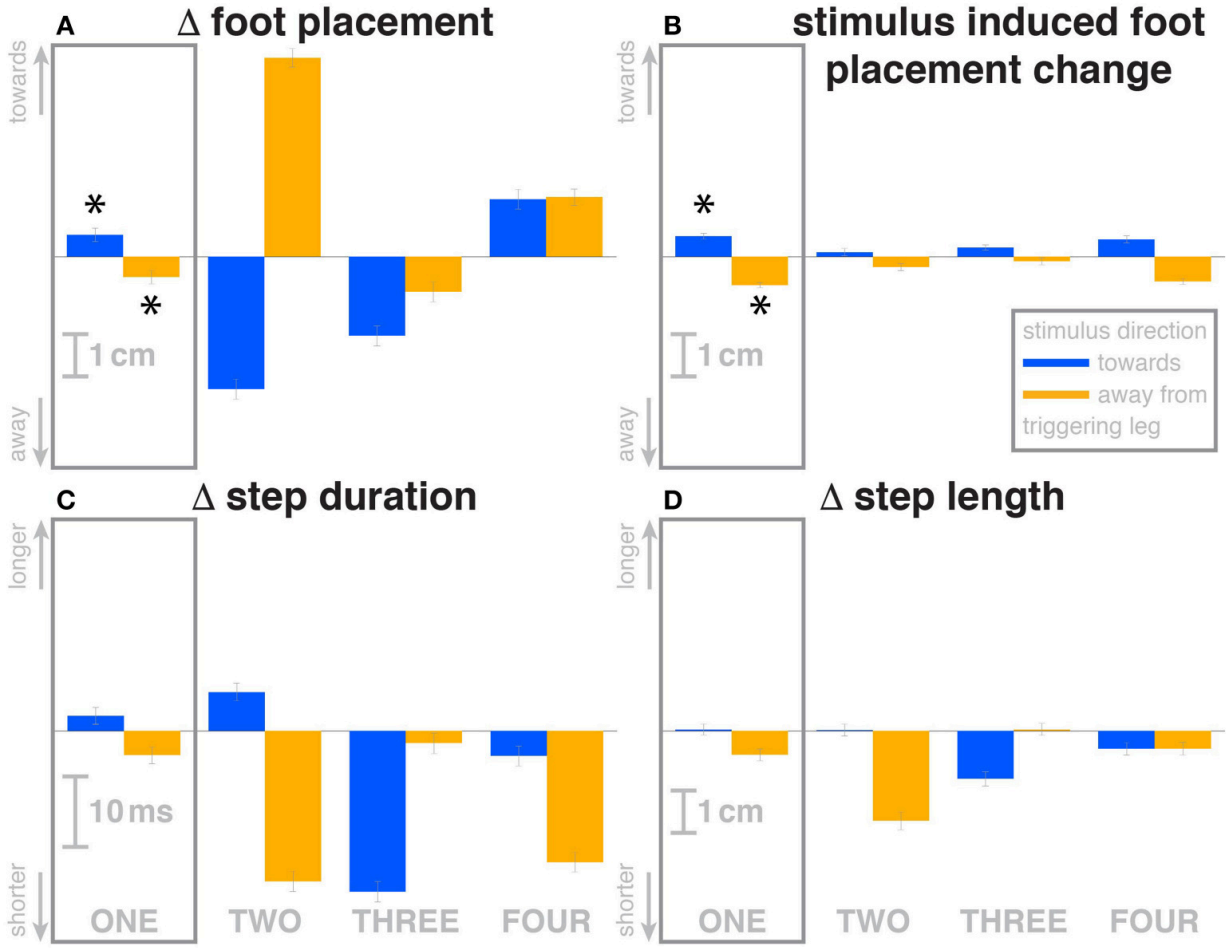
Changes in response to the visual stimulus in the medial-lateral foot placement **(A)**, step duration **(C)** and step length **(D)**, and the model-based estimate of the component due to the visual perturbation alone **(B)**. The baseline represents the average of the control steps. Shown are data for the four steps following each visual perturbation, with the first step marked for clarity. Blue bars correspond to fall stimuli *toward* the triggering leg, orange bars to fall stimuli *away* from the triggering leg. Asterisks indicate statistically significant difference from zero (see Table 3). Lack of marks indicate that no hypothesis was statistically tested.

**Figure 4:**
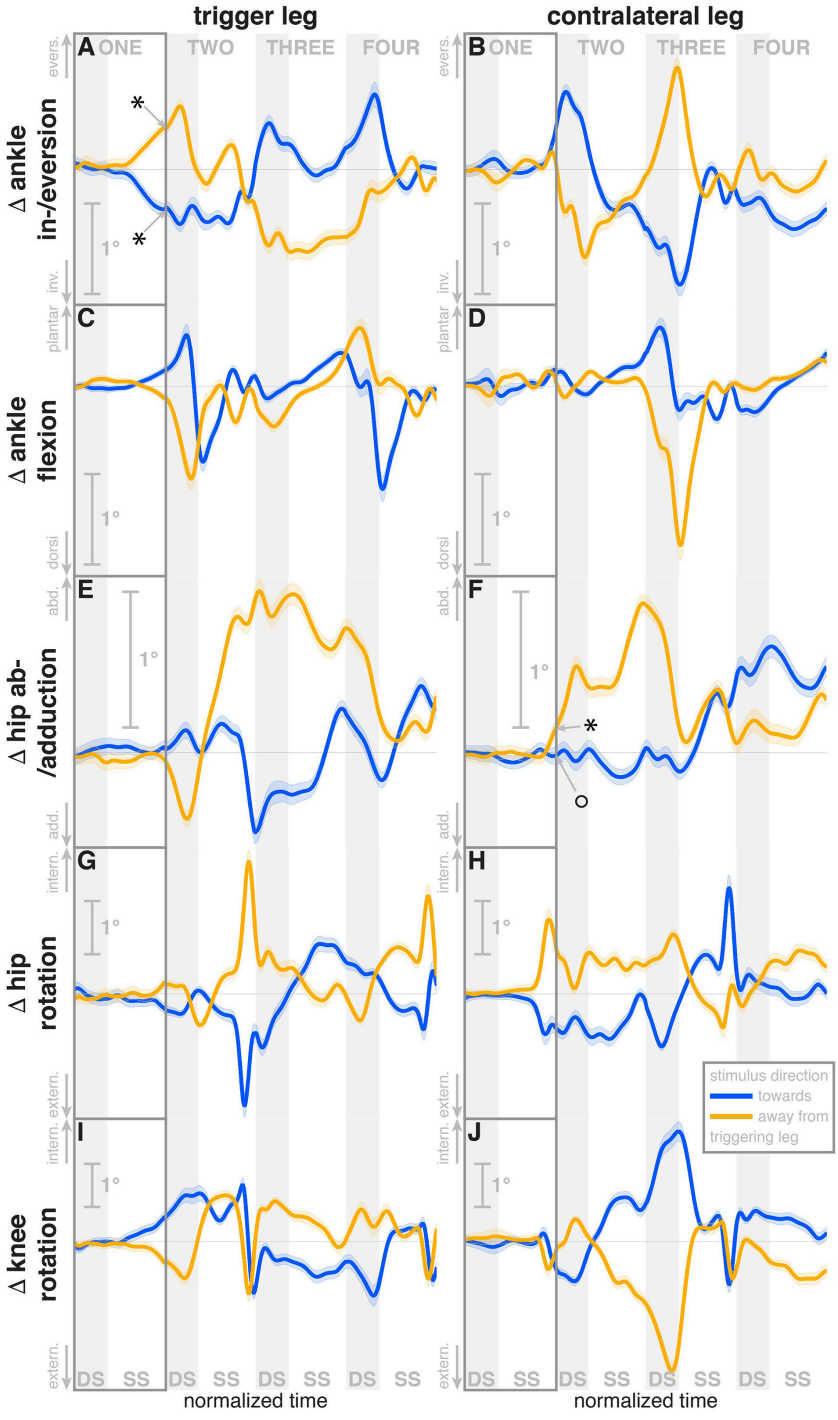
Changes in response to the visual stimulus in leg joint kinematics. The baseline represents the average of the control steps. Curves start at the heelstrike triggering the stimulus and show the subsequent four steps in normalized time, with the first step marked for clarity. Blue curves correspond to fall stimuli *toward* the triggering leg, orange curves to fall stimuli *away* from the triggering leg. Asterisks indicate statistically significant difference from zero, circles indicate lack of statistically significant difference from zero (see Table 3). Lack of marks indicate that no hypothesis was statistically tested. The shaded areas are 95% confidence intervals calculated using simplifying assumptions (see Methods).

**Figure 5:**
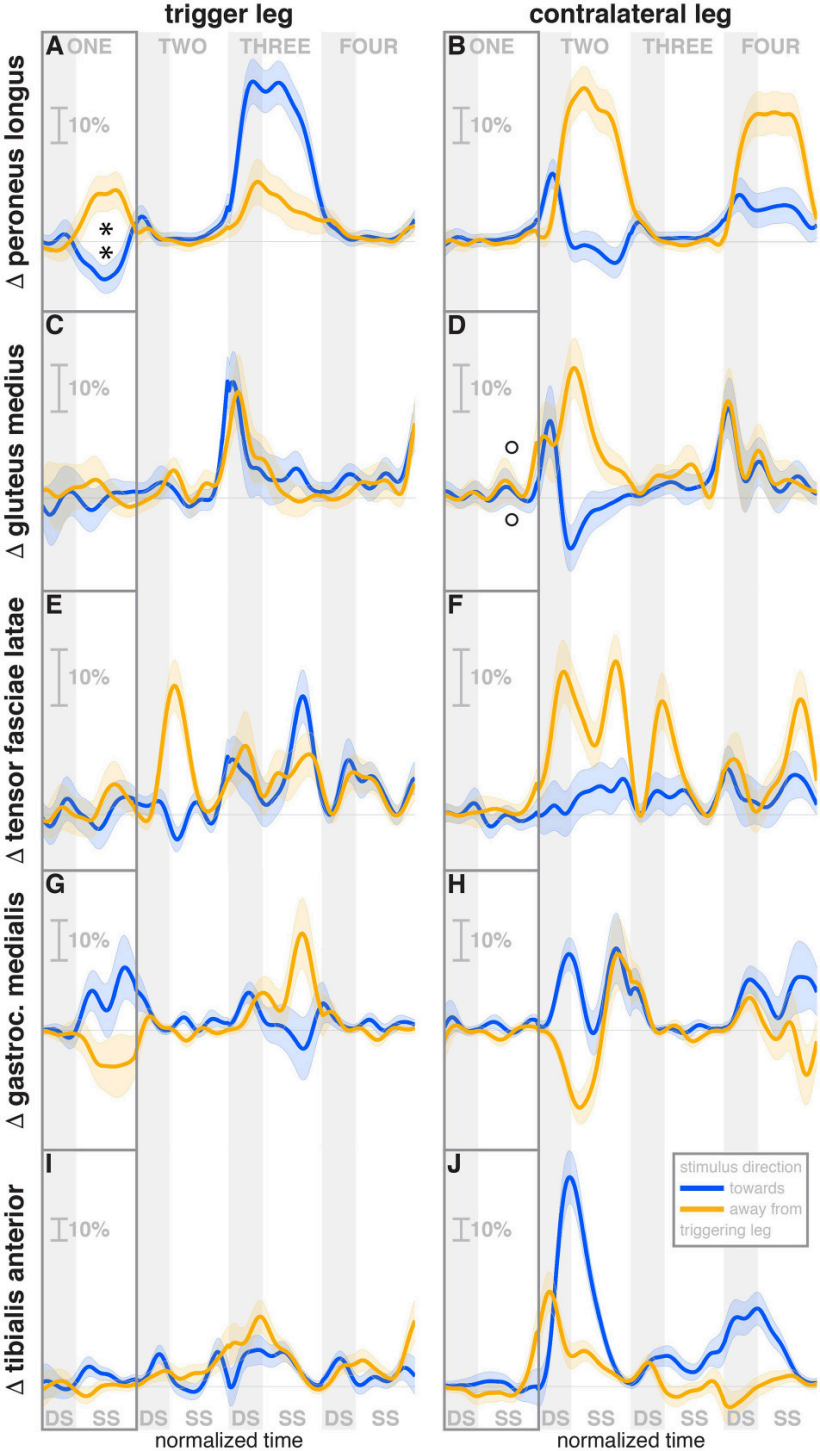
Changes in response to the visual stimulus in leg muscle EMG. The baseline represents the average of the control steps. Curves start at the heelstrike triggering the stimulus and show the subsequent four steps in normalized time, with the first step marked for clarity. Blue curves correspond to fall stimuli *toward* the triggering leg, orange curves to fall stimuli *away* from the triggering leg. Asterisks indicate statistically significant difference from zero, circles indicate lack of statistically significant difference from zero (see Table 3). Lack of marks indicate that no hypothesis was statistically tested. The shaded areas are 95% confidence intervals calculated using simplifying assumptions (see Methods). Data are normalized by rescaling to the grand average over the control steps for each subject as 100%, then subtracting the average over the control steps at each time point (see Methods).

The first post-stimulus foot placement was shifted in the direction of the perceived fall, as shown in Figure 3A. This shift was statistically significant for both stimulus directions (see Table 3). The onset of the swing heel shift in the direction of the perceived fall was estimated as 419 ms for *toward* and 447 ms for *away* stimuli (see Figure 2F). We use a model-based technique to isolate the foot placement change due to the sensory perturbation from the foot placement change that is expected from the incidental biomechanical state of the body (see Methods for details). This *stimulus-induced foot placement change* is also in the direction of the perceived fall (see Figure 3B). The magnitude of the *stimulus-induced foot placement change* is very close to the magnitude of the total foot placement, indicating that this shift is almost entirely due to the sensory perturbation. Contrary to our expectation, the swing leg hip abduction angle did not systematically change with the visual stimulus. We observed a small increase in hip abduction in step ONE for fall stimuli *away* from the current stance leg, but no corresponding hip adduction for stimuli *toward* the current stance leg (Figure 4F, Table 3). Furthermore, the onset time of this abduction change was estimated at 549 ms after stimulus, more than 100 ms later than the lateral shift of the heel marker (see Figure 2F). Similarly, the activity of the swing leg hip abductor muscles *gluteus medius* and *tensor fasciae latae* was not systematically modulated during the first step (Figures 5D,F, Table 3).

A surprising systematic change we observed was in the internal/external rotation angles of the swing leg hip and stance leg knee. Toward the end of the first step, the stance leg knee internally rotates and the swing leg hip externally rotates for fall stimuli *toward* the triggering leg (Figures 4I,H), and similar changes in the opposite direction were observed for fall stimuli *away* from the triggering leg. The onset times of the hip rotation was estimated as 450 ms for *toward* and 481 ms for *away* stimuli, those of the knee rotation as 315 ms for *toward* and 479 ms for *away* stimuli. Note that the combined effect of these two rotations on the swing foot is a lateral shift, suggesting that these, rather than hip ab-/adduction, might generate the observed foot placement response.

We also observed a systematic and comparatively large response to the visual fall stimulus in the ankle plantar-/dorsiflexion angle of the triggering leg at the end of the first post-stimulus step. For fall stimuli *toward* this leg, the ankle is more plantarflexed during the double stance phase after the first step, and more dorsiflexed for stimuli *away* from this leg (see Figure 4C). In other words, the push-off of the trailing leg is modulated to oppose the perceived fall. This push-off response starts approximately at mid-swing, estimated at 420 ms after stimulus onset for *toward* stimuli and 463 ms for *away*-stimuli, and continues through the double stance phase.

The activity of the stance leg *gastrocnemius medialis*, an ankle plantarflexor, was systematically modulated, with changes in the opposite direction as those of the *peroneus longus*. Activation decreased for fall stimuli *away* from the triggering leg and increased for stimuli *toward* the triggering leg (Figure 5G). The onset time was similar, but slightly later than that of the *peroneus longus*, estimated as 219 ms for *toward* and 258 ms for *away* stimuli. The modulation of the stance leg *tibialis anterior* activity, an ankle dorsiflexor, was very small and not systematic during step ONE.

Over the first post-stimulus step, there were small but systematic changes in step length and time in response to the visual stimulus. Both step length and time tend to be increased for *toward* stimuli and decreased for *away* stimuli (see Figures 3C,D).

### 3.2. Secondary responses

During the second post-stimulus step, the direction of the CoP change remains the same initially. After the second heel-strike, however, during the double stance phase of the third step, it crosses over to the opposite side (see Figure 2B). The ankle in-/eversion angle of the contralateral leg goes through a transient eversion when this leg enters stance at the beginning of step TWO for *toward* simuli, then inverts during single stance (see Figure 4B). For *away*-stimuli, the ankle is inverted, then everts later during single stance. The *peroneus longus* activity is changed in two different ways during the later steps. First, for *both* stimulus directions, there is a general increase in EMG for each leg when it is in stance, i.e., the trigger leg during step THREE and the contralateral leg during steps TWO and FOUR (see Figures 5A,B). Second, the magnitude of this general increase depends upon the stimulus direction relative to the current stance leg. When the stimulus direction is toward the current stance leg, muscle activation is strongly increased (blue curve in step THREE in Figure 5A and orange curves in steps TWO and FOUR in Figure 5B). When the stimulus direction is away from the current stance leg, the increase is comparatively moderate (orange curve in step THREE in Figure 5A and blue curves in steps TWO and FOUR in Figure 5B).

The foot placement of the second post-stimulus step is shifted in the direction *against* the perceived fall (see Figure 3A). This displacement is much larger than at the first step, and in the opposite direction. Foot placements THREE and FOUR are shifted, but the shift direction appears to be not dependent on the stimulus direction. The third step is shifted *away* from and the fourth step is shifted *toward* the triggering leg. In both cases, this is the direction of the swing leg making the step, so these shifts are consistent with a general increase in step width. In contrast to the total foot placement change, the direction of the *stimulus-induced foot placement change* component remains *with* the perceived fall in all cases (see Figure 3B). The magnitude is considerably reduced in steps TWO and THREE, indicating that a large part of the observed foot placement is a response to the changed mechanical state of the body. The hip of the triggering leg abducts during step TWO, when the leg is in swing, for *away* stimuli, and adducts for *toward* stimuli shortly before heelstrike. The *gluteus medius* activity of the contralateral (non-triggering) leg, which is the new stance leg in step TWO, is increased during double stance for both stimulus directions, then decreases again during single stance for *toward* stimuli, and further increases for *away*-stimuli (see Figure 5D). We did not observe any systematic modulation of *gluteus medius* activation during swing that might generate the observed foot placement change. The *tensor fasciae latae* of the triggering leg shows a systematic increase during step TWO, when it is in swing, for *away* stimuli, which could partially support the hypothesis that the strong foot placement shift in step TWO would be generated by the hip abductor muscles (see Figure 5E).

The in-/external rotation angles of the swing leg hip and stance leg knee showed a similar pattern to the first step as well, with internal rotation of the stance leg knee and external rotation of the swing leg hip for *toward* stimuli, and opposite changes for *away* stimuli (Figures 4G–J). The ankle plantar-/dorsiflexion angle of the contralateral leg shows systematic modulation during the double stance at the start of step THREE, where it is the trailing leg, similar to the triggering leg during the previous double stance. The direction dependency is also the same, with increased plantarflexion for *toward* stimuli and increased dorsiflexion for *away* stimuli (see Figure 4D). The *gastrocnemius medialis* activity retains its tendency to change in the opposite direction of the *peroneus longus* during the later steps, though the relationship is less clear (see Figures 5G,H). The *tibialis anterior* of the contralateral (non-triggering) leg shows an increase of activity during its stance period of step TWO for both stimulus directions, which tends to be larger for stimulus *toward* the triggering leg (see Figure 5J).

Changes in step time and length were systematic depending on stimulus for step TWO, with both variables increasing for *toward* stimuli and decreasing for *away* stimuli, as in the previous step (Figures 3C,D). While the directions are systematic, the amplitude is very small in some cases. For steps THREE and FOUR, both step length and time tended to decrease, regardless of stimulus direction, similar to the increase in step width for these steps.

## 4. Discussion

We studied the role of optical flow for the control of balance during walking by manipulating a virtual reality environment to give visual sensations that are indistinguishable from the optical flow experienced during an actual lateral fall. Subjects responded by accelerating their body in the opposite direction, as would be the appropriate response if the perceived fall was real. We observed the changes in muscle activation, body kinematics and ground reaction force to understand how the CNS reacts to a perceived threat to balance. Using model-based prediction allowed us to estimate the continuously ongoing control that generates corrective motor action based on the current mechanical state of the body as perceived by multiple sensory modalities, and use this estimate to isolate the motor response to the experimentally induced sensory stimulus, providing a direct estimate of the feedback loop mapping visual information to balance-related motor output. Furthermore, we studied the detailed body kinematics to determine whether humans use other, previously unknown mechanisms of balance control during walking.

The first response to the visual stimulus was the lateral ankle strategy. Early in single stance, the center of pressure under the stance leg begins to shift in the direction of the perceived fall (see Figure 2B). This goes along with a change in the in-/eversion angle of the stance leg ankle (see Figure 4A) and modulation of the stance leg *peroneus longus* muscle (see Figure 5A). The onset times of these responses are staggered, with ≈ 248 ms for the EMG, 292 ms for the CoP shift and 341 ms for the ankle angle deviation. These numbers are in the expected range of the electromechanical delay for the ankle musculature (Flevas et al., 2017), indicating that these are indeed three separate modes of observing the same phenomenon.

Toward the end of the first step, the swing foot shifts laterally in the direction of the perceived fall. Contrary to our expectations and how this mechanism is commonly explained in the literature (Kuo, 1999; Donelan et al., 2004), we did not observe a modulation of the hip abductor muscles (see Figures 5D,F) and only a small modulation of the hip abduction, but not the adduction angle to achieve this foot placement shift (see Figure 4F). Instead, our data suggest a different biomechanical mechanism of generating this foot placement shift: a combination of in-/external rotation of the stance leg knee and the swing leg hip. As the stance leg is in contact to the ground, internal rotation of the knee does not move the shank and foot, but the thigh and rest of the body instead. This occurs late in the step, when the swing leg is relatively far to the front, so the stance leg knee rotation results in a mostly lateral shift. The swing leg knee is slightly flexed, so the hip in-/external rotation shifts the heel laterally, while leaving the toe position largely invariant. Since these two rotations at the stance leg knee and swing leg hip are in opposite directions, the net effect on the yaw angle of the swing foot is relatively low.

We also observed a previously unobserved mechanism for the control of medial-lateral balance during walking. Toward the end of the first step, the stance foot ankle plantar-/dorsiflexion angle was systematically modulated depending on the visual stimulus. When subjects perceived themselves falling toward the stance foot, they increased plantarflexion in their trailing leg, and increased dorsiflexion for perceived falls away from the stance foot, generating a change in push-off force. Assuming that the direction of this push-off force difference is toward the center of mass, its effect would accelerate the body in a direction that is mostly anterior-posterior, but also partly medial-lateral. This coupling between directions is unique to the push-off mechanism, since for both the ankle strategy and the foot placement strategy, these two directions are in principle completely decoupled (Collins and Kuo, 2013). We hypothesize that in order to generate a relatively small medial-lateral acceleration of the CoM, the CNS accepts the undesired anterior-posterior acceleration as a collateral effect. This notion is supported by the modulation of step length in the following step: a stronger push-off is followed by a longer step, a weaker push-off is by a shorter step (see Figures 3C,D). The medial-lateral direction is generally believed to be substantially less stable than the anterior-posterior, which makes the medial-lateral stabilization at the cost of anterior-posterior disruption a reasonable trade-off (O'Connor and Kuo, 2009).

Most of the effects we discussed are immediate responses to the visual stimulus occurring in the first post-stimulus step. Our experimental paradigm did not completely open up the control loop, however, but only altered it temporarily, which means that the changes resulting from the motor response generated by the neural controller are also sensed and fed back into the system, which leads to second-order changes. Concretely, the visual stimulus conveys the sensation of falling to one side, and the neural controller reacts invoking different mechanisms that accelerate the body to the other side. Since the originally perceived fall was illusory, this acceleration now generates an actual fall, in the form of lateral movement of the CoM (see Figure 2A). This actual fall is sensed by the CNS and acted on, using the same mechanisms as before, but in the opposite direction. This generates a cross-over effect, where at some point the reaction to the self-generated, actual fall cancels out the reaction to the illusory fall and, after that point, dominates. Such a cross-over can be observed in step TWO in several variables, but notably *not* in the *stimulus-induced foot placement change*, which has the estimated component depending on the mechanical state of the body already removed.

Another secondary effect is the general widening of the gait observed in steps THREE and FOUR. During these steps, the direction of the foot placement changes is in the direction toward the swing foot, meaning the steps are wider. This is in contrast to the first two steps, where the direction of the foot placement is determined by the visual stimulus. We hypothesize that this is a strategy to increase the general stability of the walking body. While the three mechanisms of balance control described above, lateral ankle strategy, foot placement shift and push-off strategy, are control laws that map sensory information about movement of the body in space to directed motor responses, this step widening is a non-specific response that increases the passive stability characteristics of the walking body. This general stability increase would come at the cost of increased metabolic energy consumption (Donelan et al., 2004).

Our data provided an understanding of how the central nervous system controls the upright body during walking at an unprecedented level of detail. While it was known that vision does play a role in balance control during walking (Salinas et al., 2017; Thompson and Franz, 2017), the details of that effect were unknown. At 300–309 ms, the onset time of the lateral ankle roll was in the same range as responses to vestibular perturbations during locomotion via the vestibulospinal pathway, estimated at 247 ms during gait initiation (Reimann et al., 2017). This similarity of onset times between the vestibular and visual reactions is somewhat surprising, as changes of walking behavior in response to visual stimuli generally take longer. O'Connor and Donelan (2012) modified the relationship between optical flow and walking speed, and reported that participants modulated their speed to match the visual perception after an initial onset time of 1.4±0.3 s. Matthis et al. (2015) provided and removed visual feedback about stepping targets in different time windows, and found that removing stepping targets for a foot during that foot's preceding stance had a significant effect on stepping accuracy, but that effect disappeared when the target was removed during early swing, 0.7 s or less before the step. However, humans are clearly capable of reacting faster than that to visual stimuli. Huang et al. (2015) provided optic flow from walking down a virtual hallway and avoid spontaneously opening doors, then studied subjects brain activity when avoiding these obstacles, and reported a response time of 421±39 ms between the door opening and the press of a button to trigger an evasive side-step of the virtual avatar. Logan et al. (2014) saw first significant changes in body kinematics as soon as 489, 309, and 543 ms in response to anterior-posterior visual perturbations provided at loading, midstance, and terminal stance, but pointed out that these should not be interpreted as response latencies. Evidence that neural processing of balance-related visual information might be fast comes from work by Lopez et al. (2011), who asked participants to judge the lean direction of an almost vertical line with or without a tilted visual frame. Using high-density electrical imaging, they observed first differences between the neural activity patterns with vs. without the tilted frame at ~75−105 ms post-stimulus in the right lateral temporo-occipital, and later at ~260−290 ms in bilateral temporo-occipital and parieto-occipital cortex. These differences could be the neural substrate of a dedicated, fast sub-system that processes changes in orientation of the visual field, interprets them in relation to balance, and sends descending signals to invoke appropriate motor responses, which we observed here.

It is worth to point out that estimating onset times of these very early balance responses during walking is inherently difficult, because the movement patterns of a walking human body are highly variable. The neural controller constantly monitors the state of this system and makes adjustements. Most of these adjustments are small, but over time they add up to large variability in the state space describing the whole body. The initial, small balance responses are hidden within this host of other small balance responses occurring naturally. We provided a rough estimate of onset time here using a linear fit to the population reponse, following (Oostwoud Wijdenes et al., 2014). A more reliable determination of onset time that accounts for variability between subjects would require more data to consistently estimate the onset time of the balance response for a single subject. Either more data or a larger perturbation is necessary for this.

Our second major finding is the discovery of the push-off response. Previous research in balance control during locomotion has mostly focused on the foot placement mechanism (Townsend, 1985; Kuo, 1999; Hof, 2008; Wang and Srinivasan, 2014), with some considerations of the lateral ankle mechanism (Hof et al., 2010; Perry and Srinivasan, 2017; Reimann et al., 2017; Hof and Duysens, 2018). The possibility of a push-off response was implicated by Collins et al. who showed in model simulations that modulation of ankle push-off torque can assist in stabilization of a limit cycle walker (Kim and Collins, 2013), and later used feedback from the lateral CoM state to modulate the push-off force of human subjects wearing an emulated ankle prosthesis, resulting in increased stablity at decreased metabolic cost (Kim and Collins, 2015). The push-off mechanism would also be a functional link between balance in the medial-lateral and the anterior-posterior directions, which are traditionally seen as decoupled (Collins and Kuo, 2013). One response to Galvanic vestibular stimulation during walking (Blouin et al., 2011) is a strong modulation of the *tibialis anterior* and *gastrocnemius medialis* muscles. This was puzzling, since these two muscles act mainly in the sagittal plane, but the push-off modulation provides a clear explanation for this phenomenon. Modulating the push-off during double stance to control medial-lateral balance would adversely affect balance in the anterior-posterior direction, requiring additional control action in this direction. The small but systematic modulation of step length that we observed in response to the stimulus during the later steps might be such a secondary control action.

In conclusion, the results presented here establish a baseline of how healthy humans maintain their balance during locomotion. This is a necessary and important step in understanding how the control system breaks down in different situations. The presented results will allow the formulation of specific questions regarding the deterioration of these three balance mechanisms with advancing age and neural disorders affecting movement, such as Parkinson's disease or cerebral palsy.

## Data availability statement

The datasets generated for this study can be found at datadryad.org, doi: 10.5061/dryad.bn960cq.

## Author contributions

HR, TF, and JJ designed the experiment. HR, TF, and ET performed the experiment and analyzed the data. HR, TF, and JJ wrote the manuscript.

### Conflict of interest statement

The authors declare that the research was conducted in the absence of any commercial or financial relationships that could be construed as a potential conflict of interest.
